# Do soil microbes regulate citrus fruit quality through soil nutrient availability?

**DOI:** 10.3389/fpls.2026.1778663

**Published:** 2026-02-13

**Authors:** Dou Tang, Yuzhou Zhou, Haojie Cui, Wenjuan Liao, Pei Liu, Weijun Zhou, Sanan Nie

**Affiliations:** College of Resources, Hunan Agricultural University, Changsha, China

**Keywords:** citrus sinensis, co-occurrence network, fruit quality, microbial communities, soil nutrient

## Abstract

The relationship between soil microbes and citrus fruit quality is not fully understood at the moment. In this study, we collected citrus fruit and soil samples from fifteen orchards with generally similar planting conditions and soil profiles. Fruits were categorized into three quality types, namely, SL (small-weight and low Vitamin C), SH (small-weight and high Vitamin C), and BH (big-weight and high Vitamin C), respectively, based on PCA analysis. The results indicated significant differences (*P* < 0.05) were observed in the relative abundances of Acidobacteriia, Deltaproteobacteria, and Tremellomycetes at class level. Bacterial α-diversity showed no significant differences, whereas fungal communities exhibited significant differences in Shannon and Simpson index. Significant differences in β-diversity were observed among the groups. Microbial co-occurrence network analysis revealed a higher proportion of positive correlations in the BH group, suggesting stronger microbial cooperation. Redundancy analysis (RDA) demonstrated that fruit weight was influenced by soil pH, organic matter, and alkali-hydrolysable nitrogen as well as fungal Shannon and Simpson indices. Soil microbial taxa and available phosphorus significantly affected fruit quality indicators such as Vitamin C (Vc) content, titratable acidity, and soluble solids content. We propose that the diversity, composition, and co-occurrence networks of soil microbiota collectively influence soil nutrient availability. This nutrient availability, in turn, acts as a key determinant of citrus fruit quality.

## 1 Introduction

Citrus is one of the world’s most economically important fruit crops (Zhong and Nicolosi, 2020). In China, a major citrus producing region, the industry is a pillar of the rural economy. However, the current growth model often relies on expanding cultivation areas, leading to challenges such as stagnant yield per unit area, inconsistent fruit quality, and reduced market competitiveness (Mukhametzyanov et al., 2024). Therefore, improving fruit quality has become a critical strategy not only for increasing growers’ income but also for ensuring the sustainable development and industrial upgrading of the citrus sector (Mukhametzyanov et al., 2024).

Citrus fruit quality is governed by a complex interplay of genetic factors, ecological factors (e.g., light, water, temperature), and soil factors (Conti Taguali et al., 2025; Ramana et al., 1981). Considerable studies have focused on optimizing soil physicochemical properties through management practices such as precision fertilization (Ren et al., 2022), plastic mulching (Gao et al., 2023), and cover cropping (Wang et al., 2023) to enhance fruit quality. These practices aim to adjust key parameters like soil pH, organic matter, and the availability of macro- and micronutrients, which are indispensable for plant growth and well-known for their crucial roles in plant enzyme activities, chlorophyll synthesis, and overall metabolism (Zhou et al., 2019), making them pivotal for crop quality enhancement.

The direct management of soil chemistry presents limitations, as the availability of these nutrients to plants is predominantly regulated by biological processes. Soil microbial communities play indispensable roles in agricultural ecosystems by maintaining soil health and quality (van Wyk et al., 2017), with their compositional variations critically influencing nutrient cycling (Jiao et al., 2021; Li et al., 2021). Soil microbiomes are key components of unseen engineers of ecosystem processes, driving nutrient cycling and determining nutrient bioavailability (Hartman et al., 2018). While the importance of microbial biomass and community composition is recognized (Chang et al., 2022), previous studies often fail to capture the intricate interactions among microbial taxa that underpin ecosystem functioning. Emerging research emphasizes that microbial communities function as interconnected networks (Ge et al., 2021), and their stability and impact on plants are better predicted by co-occurrence patterns than by the presence or abundance of individual species (Jiang et al., 2024).

Recent studies in citrus systems have begun to reveal that soil microbial communities are influenced by environmental factors and are associated with tree growth (Conti Taguali et al., 2025). A systemic understanding of how the soil community structure and interaction networks link soil nutrient availability to the final fruit quality phenotype is still lacking. Most studies focus on isolated components, for instance, either soil chemistry, microbial composition or plant growth without integrating them into a coherent framework. To address this gap, we hypothesize that distinct citrus fruit quality profiles are associated with specific soil microbial community structures and co-occurrence networks, which in turn regulate the availability of key soil nutrients. This study establishes fruit quality groups based on PCA analysis from multiple fruit quality metrics across fifteen orchards. The objectives of this research are to (1) investigate the difference of soil microbial communities associated with varying citrus fruit quality, (2) illustrate the regulatory role of soil microbes and nutrients in determining fruit quality attributes. The foundation may provide novel strategies for nutrient management and soil microbiome engineering in sustainable citrus cultivation.

## 2 Materials and methods

### 2.1 Site description and soil collection

Both soil samples and fruits of Citrus sinensis were collected in the fruits harvest season of 2023 from commercial orchards in Yongxing County (26°02′–26°23′ N, 112°54′–113°20′ E), Chenzhou City, Hunan Province, China. This area is an acidic red soil region and representative citrus production area featured with a subtropical monsoon climate with mean annual temperature of 17.6°C and precipitation of 1,417 mm. Soils in the orchards were sampled using an “S” shaped transect with a five-point sampling method. Similar sized fruits without visible damage, pest infestation, or mechanical injuries were taken to ensure sample homogeneity. After collection, the samples were labelled and stored in fresh bags for preservation. To ensure a consistent genetic background, all citrus samples were the ‘Bingtang’ sweet orange cultivar. Soil samples (0-40cm depth) were immediately frozen at −80°C until DNA extraction and additional bulk soil was collected, air dried, then sieved for chemical analysis.

### 2.2 Soil properties and fruit quality analysis

Soil pH was determined potentiometrically in a 1: 2.5 soil/H_2_O suspension. Soil organic matter was analyzed by the oxidation of K_2_Cr_2_O_7_. The determine of soil alkali-hydrolysable nitrogen, available phosphorus, slowly available potassium, and available potassium was followed the description of Lu (Lu, 2000). Exchangeable calcium and magnesium were quantified by ammonium acetate extraction-inductively coupled plasma optical emission spectrometry (ICP-OES). The available sulfur iron, manganese, copper, zinc and boron were extracted by DTPA/EDTA then determine by ICP-OES (Optima 8300, PerkinElmer). The fruit quality parameters were determined according to the methods described by (Li and He, 2021). Single fruit weight was detected by gravimetry. Fruit dimensions were measured by digital caliper. Soluble solids were analyzed by hand-held refractometer. Titratable acidity was determined after NaOH titration (0.1 mol·L^−1^) addition. Vc was quantified by iodine titration (0.01 mol·L^−1^). Solid-acid ratio was calculated as soluble solids content to titratable acidity ratio.

### 2.3 DNA extraction, PCR amplification, and Illumina sequencing

Soil total DNA was extracted using MOBIO PowerSoil DNA Isolation Kit for the corresponding sample. The concentration and purity were measured using the NanoDrop One (Thermo Fisher Scientific, MA, USA). The *16S rRNA* gene of distinct regions were amplified used specific primer (806R/515F) with 12bp barcode. Primers were synthesized by Invitrogen (Invitrogen, Carlsbad, CA, USA). PCR reactions containing 25 μL of 2x Premix Taq (Takara Biotechnology, Dalian Co. Ltd., China), 1 μL of each primer (10 μM) and 3 μL DNA (20 ng/μL) template in a volume of 50 μL, were amplified by thermocycling: 5 min at 94°C for initialization; 30 cycles of 30 s denaturation at 94°C, 30 s annealing at 52°C, and 30 s extension at 72°C, followed by 10 min final elongation at 72°C. The PCR instrument was Bio-Rad S1000 (Bio-Rad Laboratory, CA, USA).

The length and concentration of the PCR product were detected by 1% agarose gel electrophoresis. Samples with bright main strip between (V4-V5: 400-450bp) can be used for further experiments. PCR products were mixed in equidensity ratios according to the Gene Tools Analysis Software (Version 4.03.05.0, SynGene). Then, mixture PCR products were purified with EZNA Gel Extraction Kit (Omega, USA). Each project selects the appropriate primers for amplification. When the final primer sequence is not known, it can be viewed in the mapping file of the analysis result package.

Sequencing libraries were generated using NEBNext^®^ Ultra™ DNA Library Prep Kit for Illumina^®^ (New England Biolabs, MA, USA) following manufacturer’s recommendations and index codes were added. The library quality was assessed on the Qubit@2.0 Fluorometer (Thermo Fisher Scientific, MA, USA) and Agilent Bioanalyzer 2100 system (Agilent Technologies, Waldbron, Germany). At last, the library was sequenced on an IlluminaHiseq2500 platform and 250 bp paired-end reads were generated (Guangdong Magigene Biotechnology Co., Ltd., Guangzhou, China).

### 2.4 Sequence data processing

Quality filtering on the paired-end raw reads was performed under specific filtering conditions to obtain the high-quality clean reads according to the Trimmomatic quality-controlled process. Purified amplicons were pooled, and pair-end sequenced on the Illumina MiSeq platform, Miseq-PE250. The raw reads were analyzed using QIIME software (version 1.9.1, http://qiime.org/) to trim off adaptors, barcodes, primers and low-quality reads. Operational taxonomic units (OTUs) were clustered at a 97% similarity threshold using the UPARSE algorithm within the USEARCH software.

### 2.5 Statistical analyses

**Figure 1:**
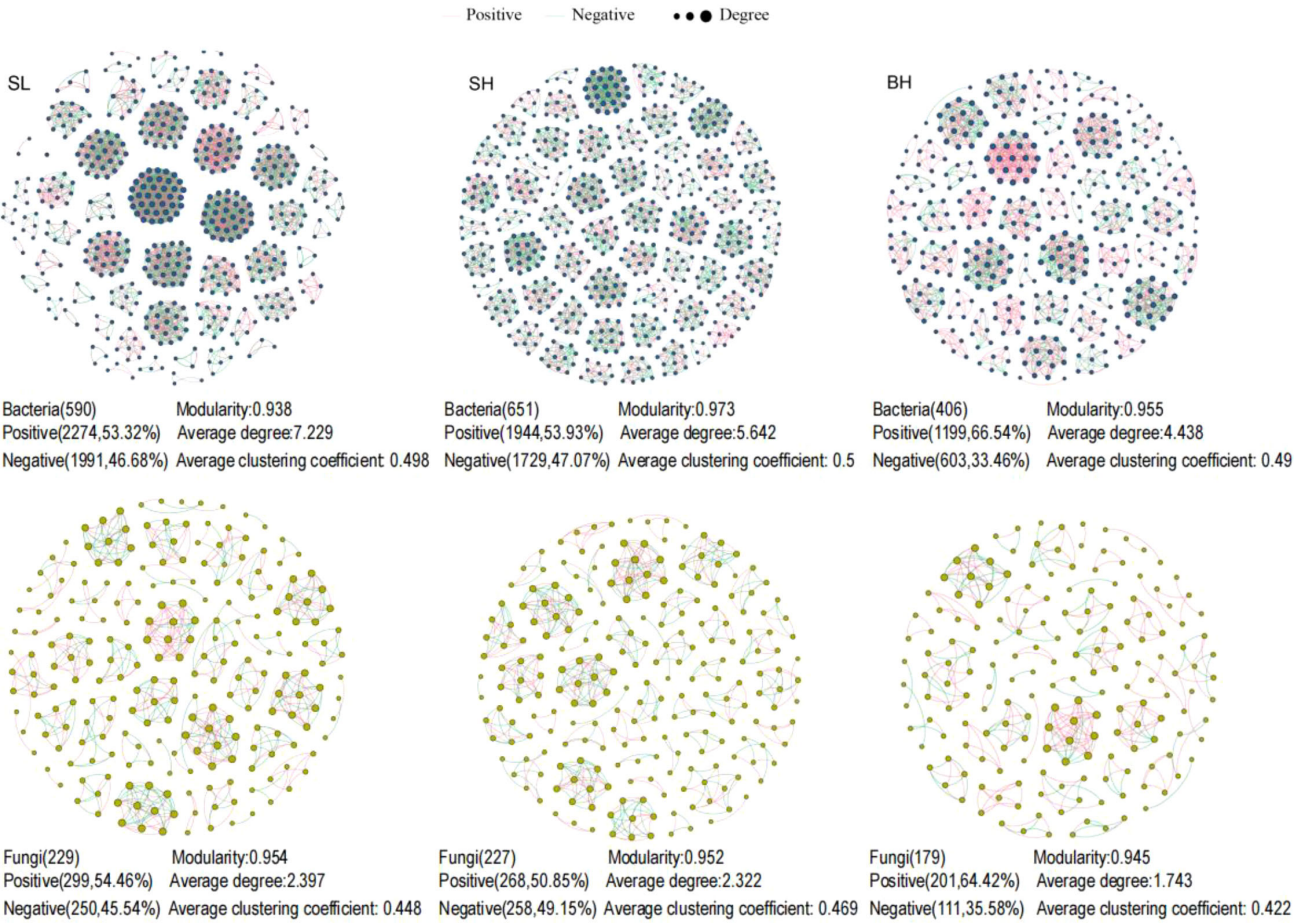
Co-occurrence network of microbial communities in different fruit quantity groups. The upper three panels represent bacteria; the lower three panels represent fungi. The edges between nodes indicate positive (red lines) and negative (green lines) correlations.

Data were analyzed using Microsoft Excel 2016, and normality tests were conducted with IBM SPSS Statistics 25. Group differences were assessed using the Duncan’s test at *p ≤* 0.05 level. Microbial co-occurrence network analysis was conducted in Gephi (v0.9.2). Based on the OTU level, after retaining taxa present in five samples, Spearman’s correlation was used to calculate relationships between microbial communities. Following threshold screening (*r*≥0.7, FDR-adjusted *p* < 0.05), a microbial co-occurrence network was constructed and visualized using Gephi, with network topology parameters calculated (**Figure 1**) (Liu et al., 2025). Redundancy analysis (RDA) examining relationships among soil microbial communities, chemical properties and fruit qualities was performed in R. The α-diversity of the bacterial communities and PCA of fruit quality were conducted in SPSS. Bray–Curtis distances of microbial community composition were computed using the vegan package in R based on OTU tables. Anosim analysis was calculated to verify the reliability of NMDS results.

## 3 Results

### 3.1 Fruit quality and its grouping by PCA

PCA was used to assess the overall differences in fruit qualities among 15 citrus orchards (**Table 1**). The results showed that the first three principal component eigenvalues ranged 1.28 to 3.25, collectively explaining 83.88% of the cumulative variance. In the rotated component matrix, the sum of the absolute values of the loadings was highest for Vc content and fruit weight, identifying them as the key discriminating variables. Based on this, the samples were categorized into three distinct groups: small fruit with low Vc (SL), small fruit with high Vc (SH), and large fruit with high Vc (BH).

**Table 1:** Principal component analysis of fruit quality variables.

Variance explained by principal components				Rotated component matrix(absolute value)				
PC	Eigenvalue	Percentage of variance(%)	Cumulative percentage(%)	Variables	PC1	PC2	PC3	Cumulative total
PC1	3.25	36.07	36.07	Vc	0.48	0.32	0.72	1.52
PC2	3.02	33.59	69.66	Weight	0.78	0.48	0.15	1.41
PC3	1.28	14.22	83.88	TSS/TA ratio	0.74	0.39	0.17	1.31
				TA	0.88	0.04	0.33	1.25
				Shape index	0.01	0.88	0.2	1.1
				LD	0.03	0.99	0.04	1.06
				TSS	0.01	0.1	0.93	1.04
				TD	0.03	0.84	0.18	1.04
				Skin thickness	0.9	0.08	0.02	1

Vc, Vitamin C content; Weight, Single fruit weight; TSS/TA ratio, Soluble solid to acid ratio; TA, Titratable acidity; LD, Longitudinal diameter; TSS, Total soluble solids; TD, Longitudinal diameter.

### 3.2 Microbial taxa and diversity in different fruit quality groups

**Figure 2:**
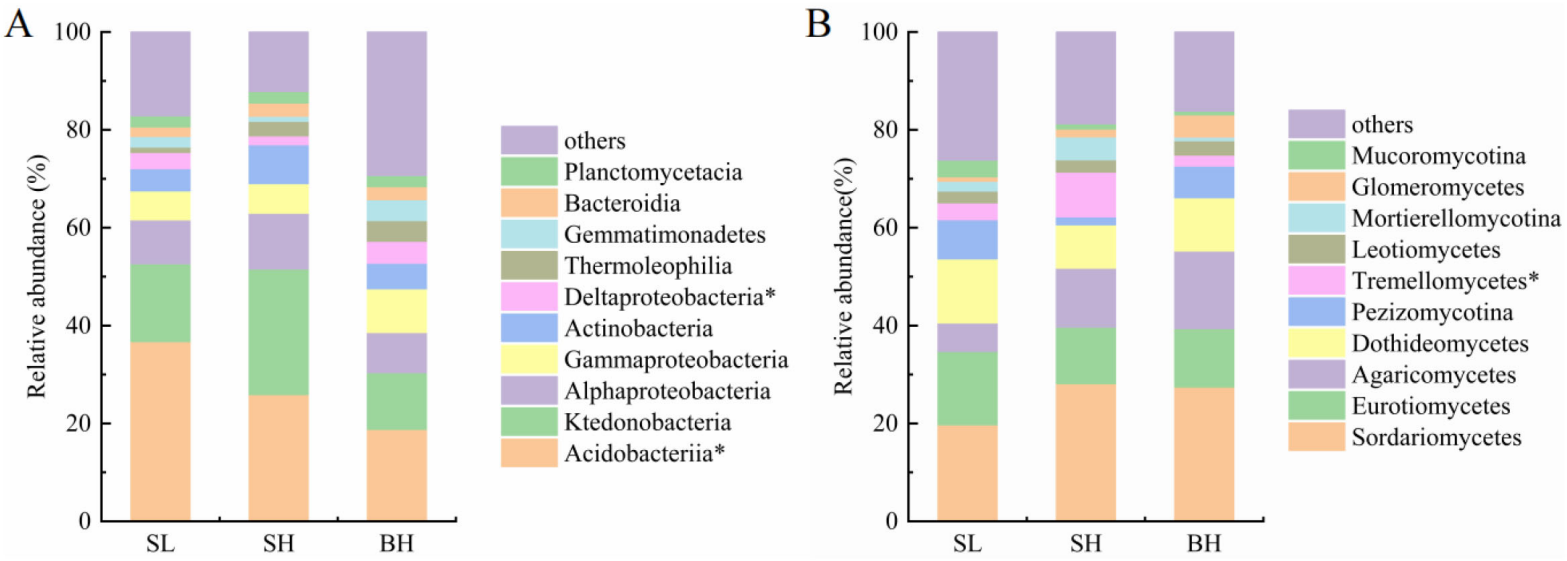
soil bacterial **(A)** and fungal **(B)** community compositions and relative abundances at class level among different fruit groups. * indicate significant differences (*p* < 0.05) in relative abundance among treatments (*n* = 5).

At the class level, taxa with a relative abundance greater than 1% were selected for differential analysis (**Figure 2**). The results indicated that the predominant bacterial classes across all treatment groups were Acidobacteriia (relative abundance of 18.78-36.71%), Ktedonobacteria (11.61-25.68%), Alphaproteobacteria (8.18-11.41%), and Gammaproteobacteria (5.91-8.95%). Compared to the SL group, the relative abundance of Acidobacteriia in the BH group decreased significantly from 36.71% to 18.78%. In contrast, the relative abundance of Deltaproteobacteria in the BH group showed a significant increase from 1.81% to 4.47% compared to the SH group. Regarding fungal communities, the major classes identified were Sordariomycetes (19.72-28.11%), Eurotiomycetes (11.57-15.01%), Agaricomycetes (5.80-15.88%), and Dothideomycetes (8.86-13.10%). The relative abundance of the class Tremellomycetes in the SH group was 9.12%, which was significantly different from that in the SL (3.45%) and BH (2.24%) groups.

No significant differences in bacterial α-diversity were detected among different groups (**Table 2**). For fungal communities, Observed species and Chao1 index showed no significant difference within three groups. However, Shannon index in BH group was higher than SL and SH groups significantly. Simpson index in BH group was higher than SL, but showed no significant difference with SH group.

**Table 2:** Diversity indices of microbial communities among treatments (*n* = 5).

Fruit quantity groups	Bacteria				Fungi			
	Observed species	Chao 1	Shannon	Simpson	Observed species	Chao 1	Shannon	Simpson
SL	1653.6± 195.77a	1924.63±242.48a	5.53±0.17a	0.98±0.01a	666.00± 30.98a	804.99± 32.86a	3.48±0.35b	0.90±0.04b
SH	1612.20±293.92a	1907.65±323.07a	5.49±0.41a	0.98±0.01a	638.20± 99.03a	787.36± 108.65a	3.68±0.11b	0.93±0.02ab
BH	1793.60±331.34a	2072.45±331.60a	5.86±0.41a	0.99±0.41a	637.80± 110.46a	759.87± 110.83a	4.12±0.39a	0.95±0.02a

Different letters in the same column indicate significant differences (p<0.05) among treatments.

**Figure 3:**
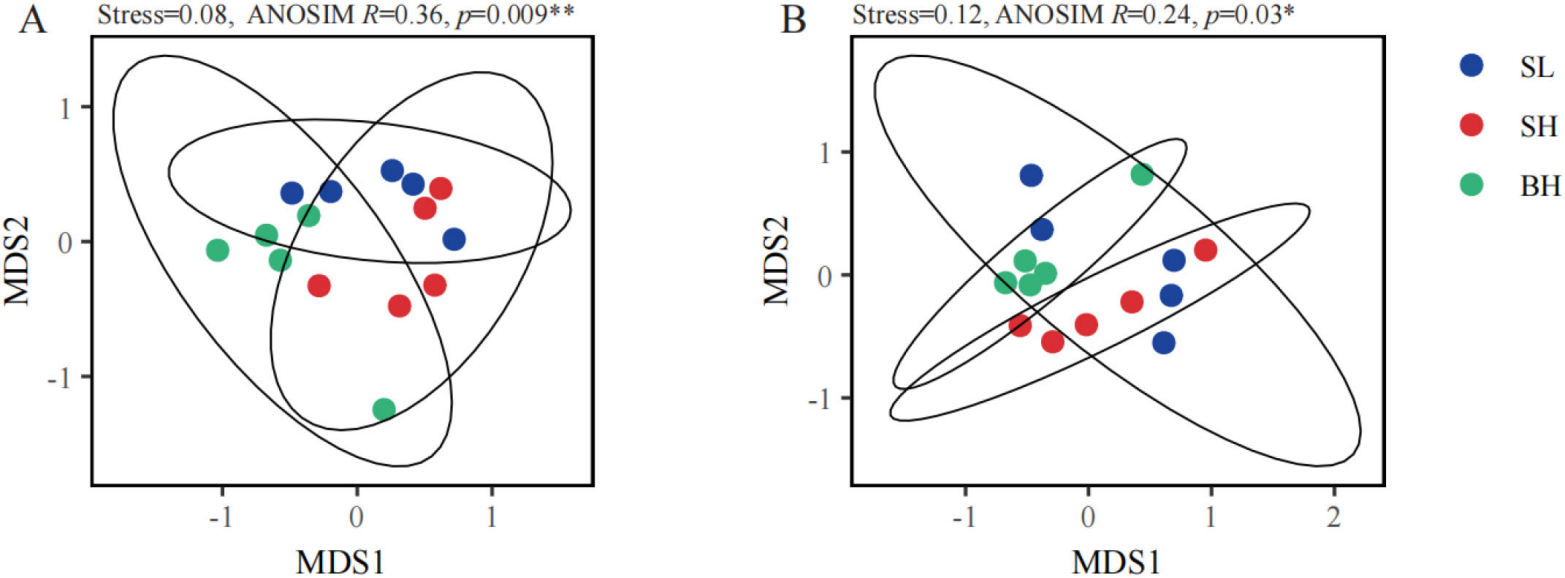
Non-metric multidimensional scaling (NMDS) of soil bacterial **(A)** and fungal **(B)** communities under different fruit quantity groups. *p < 0.05; **p < 0.01.

Non-metric multidimensional scaling (NMDS) ordination based on Bray-Curtis distances revealed significant differences in bacterial and fungal community structures among the quality groups, albeit with distinct distribution patterns (**Figure 3**). The R statistic from ANOSIM reflects the degree of separation between groups; a higher value suggests that between-group differences are greater than within-group differences. For bacterial communities, the ordination showed excellent fit (Stress = 0.08) and ANOSIM test indicated that the differences between groups were significantly greater than those within groups (ANOSIM *R* = 0.36, *p* = 0.009), suggesting a moderate separation correlated with fruit quality. In contrast, the fungal community ordination was acceptable (Stress = 0.12) and exhibited a significant yet weaker separation among groups (ANOSIM *R* = 0.24, *p* = 0.037).

### 3.3 Soil microbial co-occurrence network traits

It was indicated that the SL treatment exhibited the highest total edges (4265) and average degree (7.229) compared to the other two groups, suggesting a more tightly connected bacterial network overall. The SH treatment demonstrated the highest modularity (0.973), indicating a more complex and stable network structure. Meanwhile, the BH treatment contained the highest proportion of positive edges (66.54%), reflecting the strongest cooperative interactions among microbial communities within its network. In the fungal co-occurrence network, compared to the other two groups, the SL treatment exhibited higher nodes (229), total edges (549), average degree (2.397), and modularity (0.954) were higher than the other two groups, indicating a more tightly knit and complex fungal network structure with greater stability. The SH treatment exhibited the highest average clustering coefficient (0.469), suggesting a more highly organized and functionally modular microbial community, while the BH treatment again had the highest proportion of positive edges (64.42%). In conclusion, the microbial co-occurrence network in SL exhibited the strongest stability but poorer fruit quality, suggesting potential independence between network stability and fruit quality. The high modularity of the bacterial network in SH indicates greater functional differentiation within the bacterial community, which may promote Vc production. Although the co-occurrence network in BH showed poorer stability, enhanced cooperation between communities may be closely related to fruit quality. The connectivity and cooperativity of microbial networks may be closely related to fruit quality.

### 3.4 Relationship between soil properties, microbiota and fruit quality

**Figure 4:**
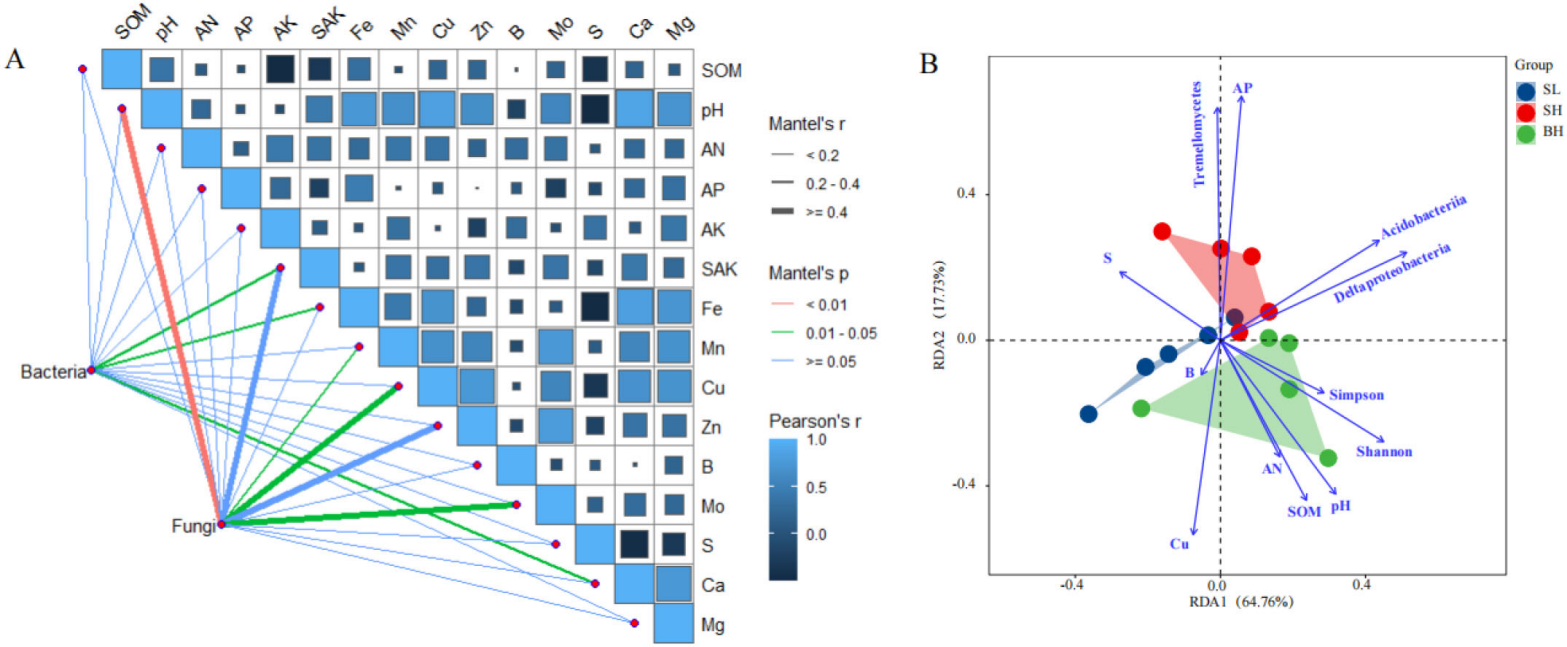
Pairwise comparisons of soil properties with a color gradient denoting Spearman’s correlation coefficients **(A)**, as well as ordination plots of the results from the redundancy analysis (RDA) to investigate correlations between soil properties, microbiota and fruit quality **(B)**.

To investigate the influence of soil physicochemical properties on microorganisms, a correlation analysis was performed between soil factors and the operational taxonomic unit (OTU) numbers of bacteria and fungi (**Figure 4A**). The results revealed that different microbial groups were driven by distinct soil factors. Bacterial OTU numbers exhibited significant correlations with slowly available potassium, available iron, and exchangeable calcium. In contrast, fungal OTU numbers were closely associated with soil pH, available manganese, available copper, and available molybdenum. And significant positive correlations were observed among pH, Fe, Mn, Cu, Zn, Mo, Ca, and Mg.

To identify the key environmental drivers of fruit quality, a redundancy analysis (RDA) was performed using soil physicochemical properties that showed significant correlations with fruit quality indicators in a prior Pearson analysis (**Figure 4B**). The RDA revealed close associations between fruit quality and both soil chemical properties and microbial community structure. The ordination model explained 82.49% of the total variance in fruit quality, with the first axis (RDA1) accounting for the majority (64.76%). In the RDA ordination space, sample points representing different fruit quality grades formed distinct and separate clusters. The distribution of the BH group was primarily driven by pH, soil organic matter (SOM), available nitrogen (AN), and the Shannon index, with its fruit quality showing a negative correlation with S content. In contrast, the SH group’s position was mainly associated with the relative abundance of Tremellomycetes and available phosphorus (AP), and its quality was negatively correlated with Cu content. Finally, the fruit quality of the SL group demonstrated negative correlations with the abundances of Acidobacteriia and Deltaproteobacteria.

Bacteria (OTU numbers of Bacteria) and Fungi (OTU numbers of Fungi) was related to each soil property by partial Mantel tests. Edge width corresponds to the Mantel’s r statistic for the corresponding distance correlations, and edge color denotes the statistical significance. SOM: soil organic matter; AN: available nitrogen; AP: available phosphorus; AK: available potassium; SAK: Slowly available potassium; Fe: iron; Mn: manganese; Cu: copper; Zn: zinc; B: Boron; Mo: molybdenum; S: sulfur; Ca: calcium; Mg: magnesium; Shannon: Shannon indices of fungal α-diversity; Simpson: Simpson indices of fungal α diversity.

## 4 Discussion

### 4.1 Effects of soil properties on microbial community of citrus orchard

Soil pH and SOM are two fundamental factors shaping the structure and function of microbial communities (Tian et al., 2021). The redundancy analysis (RDA) results in this study confirmed that soil pH is significantly positively correlated with the Shannon index of the fungal community, which directly influenced single fruit weight. Similar result showed that, in acidic soils, soil acidification directly regulated fungal community composition and specific fungal taxa, and thus indirectly affected crop yield (Ning et al., 2020). In this study, the relative abundance of Acidobacteriia in the BH group (large fruit, high Vc) soil was significantly lower than that in the SL group, which is likely related to increased pH and improved nutrient conditions, as Acidobacteriia prefers acidic, oligotrophic environments (James et al., 2022). Therefore, the abundance of certain microorganisms in citrus orchard soils may serve as an indicator of soil nutrient availability, thereby influencing fruit quality. SOM drives microbial community evolution from energy and resource substrates (He et al., 2025), not only provides carbon sources and energy for microorganisms, and its quantity and quality also determine the abundance of microbial groups (Fu et al., 2025). In this study, SOM and available nitrogen emerged as the primary environmental drivers explaining single fruit weight variation. Soil organic matter typically sustains higher microbial diversity, including richer fungal diversity, which in turn enhances land productivity through more stable nutrient cycling (Sun et al., 2022). Soil organic matter is a crucial factor in determining microbial activity, while also influencing nutrient release for both fruits and microorganisms.

Soil microorganisms are not only involved in nutrient and carbon transformations but may also shape the soil habitat through various biogeochemical and biophysical mechanisms (Wang et al., 2025; Zha et al., 2024). Microbial activities involved in the biogeochemical cycles of carbon, nitrogen, and sulfur generate protons and hydroxyl ions, thereby exerting a significant influence on soil pH (Philippot et al., 2024). The abundance of specific microbial functional groups directly correlates with nutrient transformation (Chen et al., 2022; Liu et al., 2023; Shi et al., 2024). Our study revealed an interesting association: the relative abundance of the fungal class Tremellomycetes was highest in the SH group and showed a significant positive correlation with soil available phosphorus content. This correlation raises the question of whether Tremellomycetes might contribute to the phosphorus cycle in this system. It is known that many Basidiomycota fungi, to which Tremellomycetes belong, are capable of solubilizing phosphorus by secreting organic acids or phosphatases (Jennings, 1995; Maharana et al., 2021). However, a correlation does not necessarily imply a direct causative role in phosphorus solubilization. The high abundance of Tremellomycetes could also be a consequence of higher phosphorus availability, or it may reflect other ecological functions such as interacting with phosphate-solubilizing microorganisms or occupying specific ecological niches in the rhizosphere.

Notably, correlation analysis showed that the fungal OTU number was closely related to soil available copper content. RDA indicated that the relative abundance of Tremellomycetes showed a significant positive correlation with AP, but a significant negative correlation with available copper. On one hand, organic acids secreted by phosphate-solubilizing microorganisms may alter the chemical speciation of copper in the soil through acidification of the rhizosphere microzone or complexation. On the other hand, an antagonistic interaction exists between copper and phosphorus within plants, where excessive Cu can inhibit root absorption and translocation of phosphorus (Roman et al., 2024). Therefore, the variation in Tremellomycetes abundance may serve as a microbial indicator of a complex balance between phosphorus and copper bioavailability in soil.

### 4.2 Impacts of soil nutrients characteristics on fruit quality

Soil nutrients mediate the link between soil microbial activity and fruit quality phenotypes. This study found that different nutrients drive specific aspects of external and internal fruit quality. Both AN and SOM were crucial for single-fruit weight and peel thickness, with especially strong positive correlations observed for peel thickness (**Supplementary Figure S1**). Our findings are supported by previous research. Li et al (Li et al., 2018). also demonstrated in a Ponkan orchard that nitrogen fertilizer application significantly increased fruit size and peel thickness, and found that soil nitrogen content exhibited a significant positive correlation with peel thickness. Guo et al (Xia et al., 2009). reported that within the range of nitrogen supply used, increasing N supply led to more cells per fruit and larger fruit. SOM serves not only as a nitrogen reservoir but also, by improving soil structure, enhancing water and nutrient retention capacity, and optimizing the root-zone environment, thereby indirectly yet significantly promoting the overall accumulation of plant assimilates and their partitioning to fruits (Liu et al., 2024; Zhao et al., 2025). Therefore, the optimal contents of AN and SOM in the BH group soil collectively ensured the efficient conversion of photosynthetic products into fruit biomass. We found a strong association between soil available phosphorus and vitamin C, titratable acidity, and total soluble solids. Specifically, AP exhibited a highly significant positive correlation with TSS (**Supplementary Figure S1**). Phosphorus plays a central role in plant energy metabolism, photosynthetic product transport, and sugar conversion (Liu et al., 2024; Sha et al., 2020). Ascorbic acid biosynthesis and organic acid accumulation are coupled with phosphorus-mediated respiratory metabolism and energy homeostasis (Chen et al., 2013). Consequently, AP emerges as the crucial signal determining fruit internal quality.

A specific association between Cu and fruit transverse diameter was identified in this study (**Supplementary Figure S1**), and similar positive effects of copper on fruit size have been reported (Sajid et al., 2022). Copper is a common active ingredient in fungicides routinely applied in orchards. Therefore, the observed elevated copper levels could originate from agronomic management, rather than solely from native soil pools. This exogenous input can simultaneously increase soil Cu availability and exert a strong selective pressure on the soil and rhizosphere fungal community structure. While copper is an essential cofactor for enzymes involved in cell wall lignification (Rucker et al., 1998), which could theoretically promote fruit expansion (Dauphin et al., 2022), the direct link between the microbial community (e.g., Tremellomycetes), copper bioavailability in this context, and the fruit phenotype becomes highly complex. The association we observed among copper, fungal community structure, and fruit morphology is likely a combined outcome of both nutritional and pesticide effects.

### 4.3 Association of microbial communities and fruit quality

Non-metric multidimensional scaling revealed significant differences (ANOSIM, *p* < 0.05) in soil bacterial and fungal community structures among the different fruit quality groups, providing direct evidence that microbial communities specifically influence fruit qualities. Alpha-diversity analysis revealed no significant differences in the richness and species diversity of bacterial communities among groups. In contrast, the species diversity of fungal communities was significantly highest in the BH group and was identified as the key microbial factor driving single fruit weight. This suggests that, for the citrus system in this study, the species diversity within the fungal community holds more positive significance for fruit growth than mere species number. High species diversity in the fungal community implies fewer dominant species and greater functional redundancy (Xu et al., 2025), which may ensure the stability and continuity of key ecological services such as nutrient cycling and pathogen antagonism, thereby providing a more favorable rhizosphere microenvironment for fruit development.

Regarding taxonomic composition, changes in the relative abundance of specific taxa were closely linked to quality (Jia et al., 2024; Nguyen et al., 2025). The high abundance of class Acidobacteriia in the SL group may signify a microbial strategy of high carbon efficiency but relatively conservative nutrient mineralization, consistent with impoverished soil conditions. Conversely, the significant enrichment of Deltaproteobacteria in the BH group hints at active sulfur reduction, saprotrophic metabolism, or predatory lifestyles, processes closely tied to organic matter transformation and microbial network dynamics, potentially indirectly optimizing nutrient supply.

The relationship between network topological properties and fruit quality is not simply positive. The highly connected and modular network observed in the SL group may represent a K-strategy adapted to resource-limited conditions (Wang et al., 2024). Its stability likely stems from efficient internal recycling of limited resources, with the overall ecological function geared more towards maintaining system persistence and resilience, rather than maximizing growth promotion or productivity output (Fierer, 2017). In contrast, the BH group’s network, characterized by a higher proportion of positive interactions, appears to be geared towards a more cooperative and productive regime. This cooperation-dominated network structure reflects extensive mutualistic interactions among microorganisms (e.g., cross-feeding, synergistic degradation, quorum sensing) (Liao et al., 2025; Ma et al., 2024; Newton et al., 2024). It facilitates accelerated organic matter turnover (Huang et al., 2024), promotes synchronous nutrient mineralization, and likely generates more beneficial secondary metabolites (e.g., phytohormones, siderophores), thereby creating a more favorable rhizosphere environment for plant growth (Guo et al., 2024). The rhizosphere functional trait shaped by microbial interaction patterns might be the key microbiological mechanism driving the comprehensive superior phenotype. The microbial network in the SH group exhibited high modularity but moderate cooperativity. This configuration likely supports a specific functional module (e.g., pathways related to efficient phosphorus activation and Vc precursor synthesis), enabling the achievement of high fruit quality despite smaller fruit size. Collectively, key taxa including Acidobacteriia, Deltaproteobacteria, and Tremellomycetes modulate microbial interaction networks via their distinct metabolic profiles, thereby converting environmental signals into shifts in nutrient availability that shape fruit development and quality outcomes.

## 5 Conclusions

In summary, soil properties and nutrient characteristics are fundamental in shaping specific microbial community structures, key taxa, and interaction network patterns. These microbial assemblages, in turn, regulate the efficiency of nutrient transformation and supply, ultimately determining citrus fruit quality. Therefore, enhancing soil organic matter, optimizing nitrogen and phosphorus management, and fostering microbial communities with high fungal diversity and strong cooperativity represent crucial ecological strategies for high-quality citrus production. Future research should expand sample sizes or employ controlled experiments to strengthen these findings. Furthermore, integrating multi-omics approaches, such as metagenomics and metabolomics, will be essential to elucidate the expression of key functional genes in critical microbial taxa and their associated metabolic pathways.

## Data Availability

The datasets presented in this study can be found in online repositories. The names of the repository/repositories and accession number(s) can be found below: https://www.ncbi.nlm.nih.gov/, PRJNA1291981.
