# Supplementary material for: Do soil microbes regulate citrus fruit quality through soil nutrient availability?
Source: Front Plant Sci. 2026 Feb 13;17:1778663. doi: 10.3389/fpls.2026.1778663 (PMC12946132; doi:10.3389/fpls.2026.1778663)
Supplement: Supplementary file 1 [file DataSheet1.doc]

Supplementary Table 1. Soil physicochemical properties under different fruit quality groups

|  | SL | SH | BH |
| --- | --- | --- | --- |
| SOM (g·kg^-1^) | 22.67±2.51a | 20.7±6.44a | 26.52±8.32a |
| pH (-) | 4.6±0.22b | 4.73±0.24b | 5.44±0.47a |
| AN (mg·kg^-1^) | 75.69±31.94a | 72.96±14.64a | 87.64±6.46a |
| AP (mg·kg^-1^) | 19.98±8.97a | 46.45±34.04a | 26.95±20.23a |
| AK (mg·kg^-1^) | 155.14±84.72a | 198.21±153.35a | 128.72±24.38a |
| SAK (mg·kg^-1^) | 163.57±44.56a | 190.64±69.87a | 329.34±145.97a |
| Fe (mg·kg^-1^) | 29.91±22.65a | 31.95±24.61a | 59.77±27.84a |
| Mn (mg·kg^-1^) | 15.8±16.94a | 12.14±18.72a | 30.79±16.79a |
| Cu (mg·kg^-1^) | 1.17±1.23a | 1.03±1.12a | 3.32±2.43a |
| Zn (mg·kg^-1^) | 1.35±1.01a | 2.59±3.15a | 7.99±12.31a |
| B (mg·kg^-1^) | 0.39±0.42a | 0.23±0.09a | 0.24±0.10a |
| Mo (mg·kg^-1^) | 0.18±0.17a | 0.1±0.02a | 0.3±0.30a |
| S (mg·kg^-1^) | 57.29±50.69a | 51.07±34.16a | 17.86±9.24a |
| Ca (cmol·kg^-1^) | 7.49±4.11b | 8.34±6.44b | 20.13±8.02a |
| Mg (cmol·kg^-1^) | 1.07±0.62a | 1.44±1.44a | 2.32±0.61a |


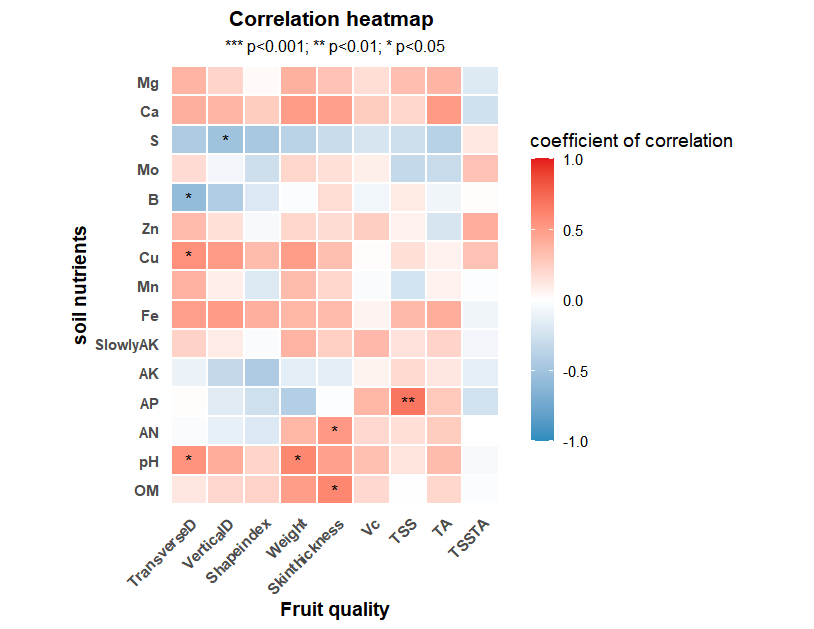


Supplementary Figure 1. Correlation heatmap between fruit quality and soil physicochemical properties
